# Effects of External Auditory Meatus Occlusion on Ocular Vestibular Evoked Myogenic Potentials Induced by Bone Conducted Sound

**DOI:** 10.3389/fneur.2021.659820

**Published:** 2021-04-13

**Authors:** Toshihisa Murofushi, Masafumi Ohki, Masahito Tsubota

**Affiliations:** ^1^Department of Otolaryngology, Teikyo University School of Medicine Mizonokuchi Hospital, Kawasaki, Japan; ^2^Department of Otolaryngology, Saitama Medical University Saitama Medical Center, Kawagoe, Japan

**Keywords:** bone conduction, oVEMP, external auditory meatus, occlusion effect, B81

## Abstract

To facilitate more reliable recordings of the ocular vestibular evoked myogenic potentials (oVEMP) induced by bone-conducted sound using the B81 bone conduction transducer, we preliminarily studied the effects of external auditory meatus occlusion using an earplug on such oVEMP. Eight healthy volunteers (four males and four females, 26–48 years of age, mean age: 34. 5 years) and 14 patients with vestibular disease (2 males and 12 females, 18–59 years of age, mean age: 41.5 years) were enrolled. oVEMP testing was performed using a B81 placed on the temple. Tone bursts (500 Hz, rise/fall time: 2 ms, plateau time: 2 ms, and 70 dB nHL) were presented at a rate of 5.1 Hz. N1-P1 amplitudes were measured and analyzed. Occlusion resulted in significantly larger N1-P1 amplitudes [mean ± SE (SD): 12.3 ± 1.67 (6.71) μV vs. 9.55 ± 1.55 (6.21) μV; *p* = 0.020, paired *t*-test]. While four patients did not exhibit any response on either side in the absence of occlusion, all of them showed unilateral or bilateral responses when occlusion was employed. In any patient occlusion did not result in loss of oVEMP responses. External auditory meatus occlusion using an earplug could allow more reliable recordings of bone conduction transducer-induced oVEMP.

## Introduction

At present, two types of vestibular evoked myogenic potentials (VEMP) are examined in the clinical setting. The first is cervical VEMP (cVEMP), which are recorded in the sternocleidomastoid muscle in order to test saccular function. The other is ocular VEMP (oVEMP), which are recorded beneath the lower eyelid in order to test utricular function (1). Although cVEMP can be easily recorded using air-conducted sound (ACS), such as 500-Hz tone bursts (2, 3), recording oVEMP using ACS is harder. For this reason, bone-conducted vibrations (BCV) have been used to record oVEMP (4). A mini-shaker (BK4810) is widely utilized to induce BCV. However, it is heavy and hard to calibrate accurately. The B71 conventional bone conduction transducer (RadioEar) was tried as an alternative. However, the output of the B71 is not always sufficient to for measuring oVEMP responses (5). A new bone conduction transducer, the B81 (RadioEar), has recently become available. The B81 has a higher output than the B71 (6). Although the B81 evokes better responses than the B71, the associated oVEMP responses are still unstable. Therefore, some ingenuity is required to obtain stable oVEMP responses using the B81.

It is known that occluding the external auditory meatus lowers the hearing threshold for bone-conducted sound. This is known as the occlusion effect (7, 8). The occlusion of the external auditory meatus improves hearing thresholds by 10–20 dB at frequencies of 250 and 500 Hz (7, 9). Therefore, occluding the external auditory meatus might improve VEMP responses. Handzel and Himmelfarb (10) reported that cVEMP responses could be augmented by occlusion. We applied this method to oVEMP recording in order to obtain stable oVEMP responses using the B81.

Herein, we preliminarily report the effects of occlusion of the eternal auditory meatus on the bone-conducted (BC) oVEMP responses of healthy subjects and patients with vestibular disease, and discuss the significance of the occlusion method for BC oVEMP recording in the clinical setting.

## Subjects and Methods

### Subjects

Eight healthy volunteers (four males and four females, 26–48 years of age, mean age: 34.5 years) and 14 patients with vestibular disease (2 males and 12 females, 18–59 years of age, mean age: 41.5 years) were enrolled. The diagnoses of the 14 patients with vestibular disease included Meniere's disease in seven cases (two definite and five probable) (11), delayed endolymphatic hydrops (ipsilateral type) in two cases (12), vestibular migraine in two cases (one definite, one probable) (13), and vestibular neuritis in three cases (14). None of the patients had conduction problems in the external or middle ear. The healthy volunteers did not have a medical history of vertigo or hearing loss.

### Methods

oVEMP were measured using the Eclipse system (Interacoustics, Middelfart, Denmark). Active electrodes were placed just beneath the lower eyelid while a reference electrode was placed on the chin. The ground electrode was placed on the nasion. Tone bursts (500 Hz, rise/fall time: 2 ms, plateau time: 2 ms, and 70 dB nHL) were presented at a rate of 5.1 Hz using a B81 bone conduction transducer (Radioear, New Eagle, USA). The bone conduction transducer was fitted with the standard steel spring headband and placed on the area just anterior to the helix at the level of the eyes. For the recordings obtained beneath the right (left) eye, the bone-vibrator was placed on the preauricular area on the opposite side to the recording site. Placement of the transducer was performed to be symmetrical as accurately as possible. One hundred responses were bandpass-filtered (10–1,000 Hz) and averaged. During the recordings, the subjects were asked to maintain a 20-degree upward gaze. To confirm the reproducibility of the results, two runs were performed for each ear. The N1-P1 amplitude was measured, and the mean of two runs was used. In addition to the recordings obtained without occlusion of the external auditory meatus, recordings involving occlusion of the external auditory meatus, which was achieved using an earplug made of urethane foam, were obtained. Recordings with and without occlusion were performed in random order. Insertion and removal of an earplug were performed without touching the transducer. The placement site was chosen to enable an examiner to insert or remove an earplug without touching the transducer.

To assess the interaural amplitude difference, the asymmetry ratio (AR) was calculated as follows:

AR = 100 × (Alarge – Asmall)/(Alarge + Asmall)

Alarge: N1-P1 amplitude on the side on which the N1-P1 amplitude was larger.

Asmall: N1-P1 amplitude on the side on which the N1-P1 amplitude smaller.

Informed consent was obtained from each subject. This study was approved by the ethics committee of Teikyo University School of Medicine (TR 20-078).

## Results

### Responses of Healthy Subjects

**Figure 1:**
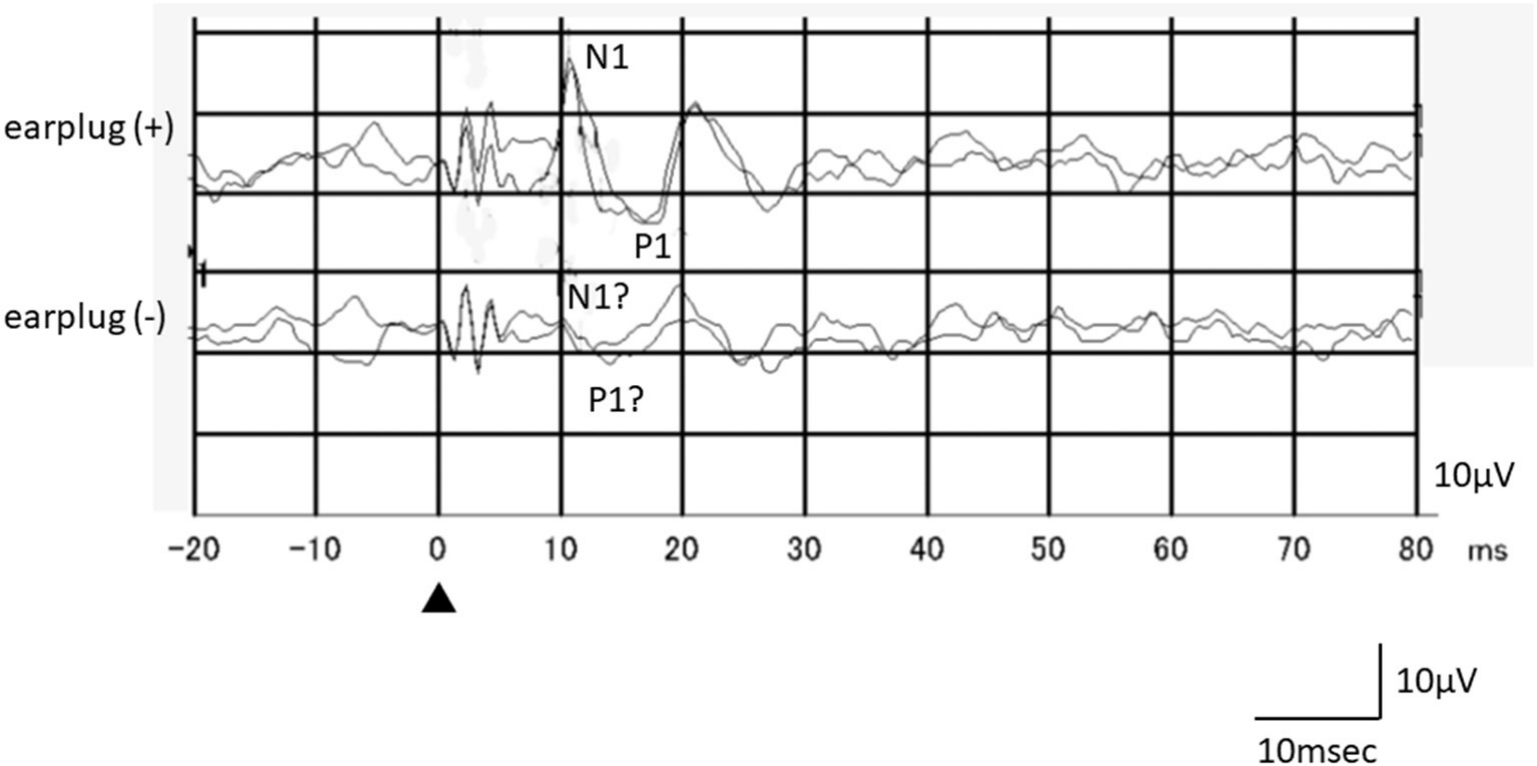
An example of the responses of a healthy subject (a 26-year-old female, left side stimulation). Although oVEMP responses in the recording obtained without occlusion were not clear, she exhibited reproducible clear oVEMP responses in the recording obtained with occlusion. Recording to the left side stimulation was performed beneath the right eye.

**Figure 2:**
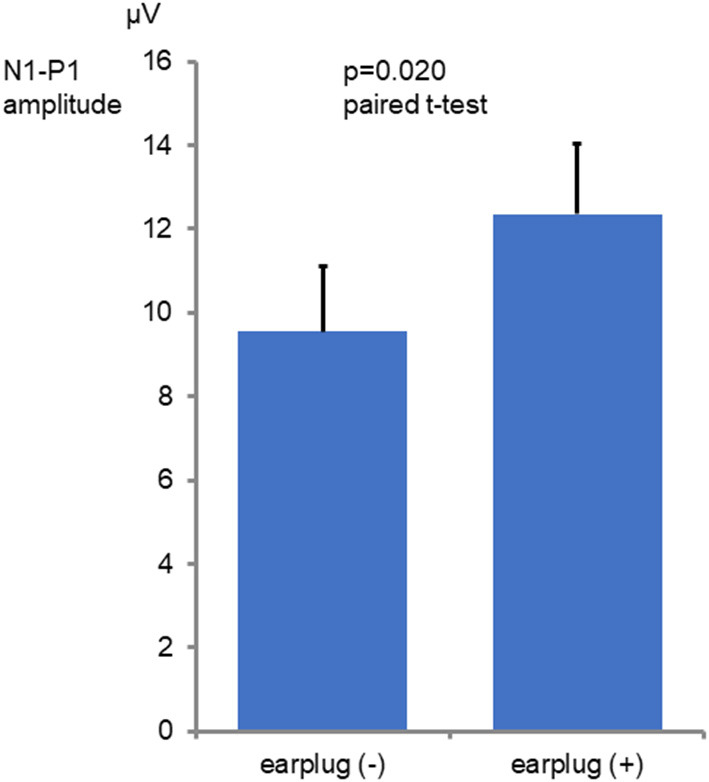
Comparison of the N1-P1 amplitude values seen with and without occlusion. The graphs show mean and SE values. The N1-P1 amplitude was significantly larger in the with occlusion conditions than in the without occlusion conditions (*p* = 0.020 paired *t*-test).

Among the 16 ears of the 8 healthy subjects, 15 showed responses in the recordings obtained without occlusion, while all 16 ears exhibited responses in the recordings obtained with occlusion (Figure 1). The N1-P1 amplitude was significantly larger in the recordings obtained with occlusion [mean ± SE (SD): 12.3 ± 1.67 (6.71) μV] than in those obtained without occlusion [9.55 ± 1.55 (6.21) μV] (mean difference: 2.75 μV, *p* = 0.020, paired *t*-test; Figure 2). The mean AR was 21.4 ± 5.52 (15.6) and 32.3 ± 11.6 (32.8) in the presence and absence of occlusion, respectively.

### Responses of Patients

**Figure 3:**
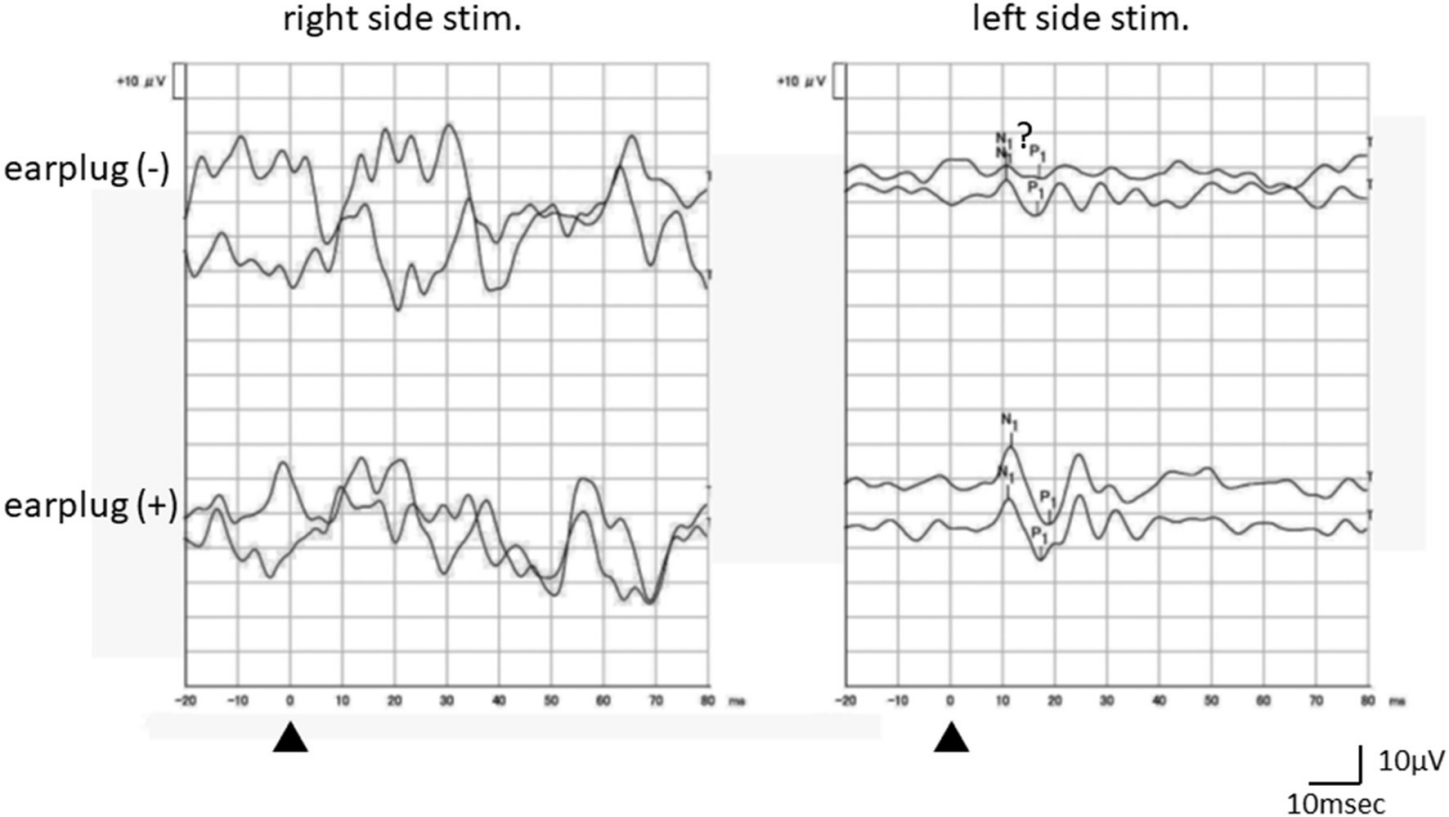
An example of the responses of a vestibular patient (a 36-year-old female, right probable Meniere's disease). Although oVEMP responses without occlusion were not clear enough, the recording obtained with occlusion showed clear reproducible responses on the left side stimulation. No response was obtained on the right side stimulation even with occlusion. Recording to the right (left) side stimulation was performed beneath the left (right) eye.

The results of the vestibular patients are summarized in Table 1. Four patients did not exhibit any response on either side in the absence of occlusion. Of these four patients, two showed bilateral responses and two demonstrated unilateral responses when the recordings were performed with occlusion (Figure 3).

**Table 1:** Summary of the patients' results.

		**Earplug (+)**			**Total**
		**Bilaterally absent**	**Unilaterally absent**	**Bilaterally present**	
	**Bilaterally absent**	0	2	2	4
**Earplug (–)**	**Unilaterally absent**	0	4	1	5
	**Bilaterally present**	0	0	5	5
	**Total**	0	6	8	14

Five patients only showed unilateral responses in the recordings obtained without occlusion. Among them, four patients still only showed unilateral responses in the recordings obtained with occlusion. All of the patients that exhibited bilateral responses in the recordings obtained without occlusion also demonstrated bilateral responses in the recordings obtained with occlusion.

## Discussion

This preliminary study showed that occlusion of the external auditory meatus produced a significant increase in the N1-P1 amplitudes of BC oVEMP recorded with the B81 in healthy subjects. The standard error (deviation) of N1-P1 amplitude obtained with occlusion was smaller than that obtained without occlusion. These findings mean that performing oVEMP tests with occlusion results in a narrower normal range than conducting them without occlusion. Large SE (SD) would be due to unclear (almost absent) responses. This would probably lead to an improvement in the sensitivity of such testing.

The site of the stimulator is very important for recording of oVEMP. Therefore, we tried to place it on the same place as accurately as possible. The site of the B81 bone conduction transducer is different from the usual site. This place was chosen to do all recording without touching the transducer. The site of the reference electrode is also unusual. It is due to the limitation of the device. However, these factors will not affect the results of this study, the superiority of BC oVEMP with occlusion to without occlusion.

As for the patients with vestibular disease, 4 of the 14 patients showed no oVEMP responses on either side in the recordings obtained without occlusion. Among them, two patients showed unilateral responses and two patients demonstrated bilateral responses in the recordings obtained with occlusion. In other words, the bilateral absence of oVEMP responses in tests performed without occlusion seems to be a false positive. These results suggest that occluding the external ear canal improves the reliability of oVEMP testing performed using the B81 bone conduction transducer.

The fact that occluding the external auditory meatus lowers the hearing threshold for BC sound has been known as the occlusion effect since the 19th century (7). The occlusion of the external auditory meatus improves hearing thresholds by 10–20 dB at frequencies of 250 and 500 Hz (7, 9). As 500-Hz tone bursts are usually used for recording VEMP, occlusion of the external ear canal seems to be beneficial for inducing VEMP responses although it depends on the mechanism underlying the occlusion effect.

Rotem Betito et al. (9) and Handzel and Himmelfarb (10) reported that external auditory meatus occlusion improved the BC cVEMP responses of healthy subjects. Rotem Betito et al. also found that the effect of occlusion on BC cVEMP was not observed in ears filled with water, but was seen in ears that had been occluded using earplugs. This suggests that earplugs may prevent bone vibrator-induced sound pressure in the external auditory meatus from escaping and reinforce the sound pressure transmitted to the inner ear.

We assumed that occlusion of the external auditory meatus using an earplug could also facilitate the recording of BC oVEMP induced using bone conduction transducers, such as the B71 and B81. Although the outputs produced by the B81 are stronger than those produced by the B71 (6), they are not always sufficient to produce stable oVEMP responses. The preliminary results of the current study support our assumption. It might be beneficial to add occlusion of the external auditory meatus to the protocol for BC oVEMP recordings performed using the B81.

There are some limitations in this study. This is a small-sized pilot study. The significance of the external auditory meatus should be confirmed in a larger study. Then, the relationship between the site of BC stimulation and the extent of occlusion effect should be also investigated because the stie of BC stimulation has large effects for amplitudes (15, 16). Then, the occlusion effect in midline stimulation will be investigated. Asymmetry of occlusion might have some effects. This point should be also studied in the future study.

## Data Availability Statement

The raw data supporting the conclusions of this article will be made available by the authors, without undue reservation.

## Ethics Statement

The studies involving human participants were reviewed and approved by the ethics committee of Teikyo University School of Medicine (TR 20-078). The patients/participants provided their written informed consent to participate in this study.

## Author Contributions

TM wrote the manuscript. MO and MT reviewed and edited the manuscript. All of the authors contributed extensively to the work presented in this paper. All of the authors contributed to the data collection.

## Conflict of Interest

The authors declare that the research was conducted in the absence of any commercial or financial relationships that could be construed as a potential conflict of interest.
